# A Review of Thermal Property Enhancements of Low-Temperature Nano-Enhanced Phase Change Materials

**DOI:** 10.3390/nano11102578

**Published:** 2021-09-30

**Authors:** Joseph D. Williams, G. P. Peterson

**Affiliations:** Georgia Institute of Technology, Woodruff School of Mechanical Engineering, Atlanta, GA 30332, USA; bud.peterson@gatech.edu

**Keywords:** nano-enhanced PCM, phase change materials, thermal conductivity, latent heat, thermal energy storage

## Abstract

Phase change materials (PCMs) are of increasing interest due to their ability to absorb and store large amounts of thermal energy, with minimal temperature variations. In the phase-change process, these large amounts of thermal energy can be stored with a minimal change in temperature during both the solid/liquid and liquid/vapor phase transitions. As a result, these PCMs are experiencing increased use in applications such as solar energy heating or storage, building insulation, electronic cooling, food storage, and waste heat recovery. Low temperature, nano-enhanced phase change materials (NEPCM) are of particular interest, due to the recent increase in applications related to the shipment of cellular based materials and vaccines, both of which require precise temperature control for sustained periods of time. Information such as PCM and nanoparticle type, the effective goals, and manipulation of PCM thermal properties are assembled from the literature, evaluated, and discussed in detail, to provide an overview of NEPCMs and provide guidance for additional study. Current studies of NEPCMs are limited in scope, with the primary focus of a majority of recent investigations directed at increasing the thermal conductivity and reducing the charging and discharging times. Only a limited number of investigations have examined the issues related to increasing the latent heat to improve the thermal capacity or enhancing the stability to prevent sedimentation of the nanoparticles. In addition, this review examines several other important thermophysical parameters, including the thermal conductivity, phase transition temperature, rheological affects, and the chemical stability of NEPCMs. This is accomplished largely through comparing of the thermophysical properties of the base PCMs and their nano-enhanced counter parts and then evaluating the relative effectiveness of the various types of NEPCMs. Although there are exceptions, for a majority of conventional heat transfer fluids the thermal conductivity of the base PCM generally increases, and the latent heat decreases as the mass fraction of the nanoparticles increases, whereas trends in phase change temperature are often dependent upon the properties of the individual components. A number of recommendations for further study are made, including a better understanding of the stability of NEPCMs such that sedimentation is limited and thus capable of withstanding long-term thermal cycles without significant degradation of thermal properties, along with the identification of those factors that have the greatest overall impact and which PCM combinations might result in the most significant increases in latent heat.

## 1. Introduction

### 1.1. Phase Change Materials

Since the energy crisis of the early 1970s [1], there has been an increased focus on thermal energy storage (TES) materials with a particular focus on phase change materials (PCMs), due to their ability to store sizable amounts of thermal energy. These PCMs have been categorized into three main types: organic, inorganic, and eutectic. Organic PCMs consist primarily of paraffin waxes, ethylene glycols (polymers), and fatty acids [1,2,3,4]. Inorganic PCMs typically consist of hydrated salts and some metallic substances [1,3,4,5], and a majority of the time have a larger range of phase change temperatures and greater latent heat capabilities, when compared to that of most organic PCMs [6]. Eutectic PCMs can be further classified as organic or inorganic PCMs or sometimes a combination of both organic and inorganic. Typically, eutectic PCMs have a greater utility, due to the ability to modify/control the freezing temperature, when compared with single-compound PCMs [3,7]. The ability to modify the size and range of the phase change (freezing) temperature has proven to be of considerable value, and hence, interest. Considered together, these three types of PCMs are currently under consideration for potential applications such as building insulations, water heating, food storage, solar energy harvesting, waste heat recovery, electronic applications [4], and more recently, applications related to the shipment of cellular-based materials and vaccines, both of which require precise temperature control for sustained periods of time.

**Figure 1:**
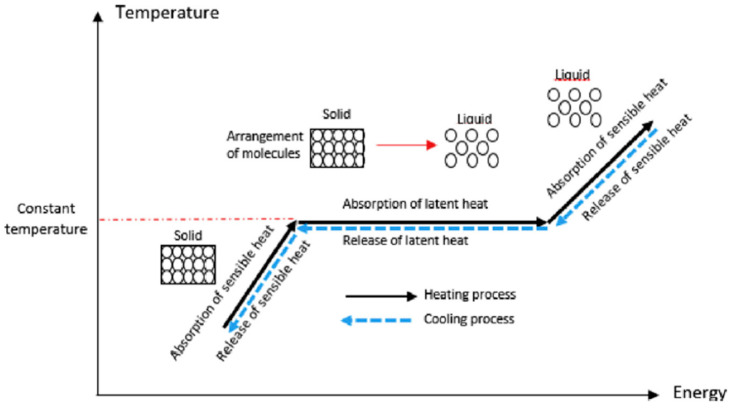
Temperature to energy absorption/release relation of a PCM. (Reprinted with permission from ref. [3]. Copyright 2019 Journal of Energy Storage).

As implied by the name, the high energy storage capacity of phase change materials is a result of the change in internal energy and specific heat capacity, occurring at constant temperature, while undergoing a change of phase (i.e., solid–liquid, liquid–vapor, and solid–vapor). This process of energy storage can be classified into two categories: the sensible heat and latent heat storage. These two components can be represented in their most basic form as:

(1) $$Q=\overset{T_{m}}{\underset{T_{i}}{\int }}mC_{p}dT+ma\Delta H+\overset{T_{f}}{\underset{T_{m}}{\int }}mC_{p}dT$$

where *Q* is the heat transfer, *m* is the mass of the PCM, $C_{p}$ is the specific heat, a is the percentage of the mass, *m*, that is undergoes a phase transition, i.e., melting and ΔH, which represents the change in enthalpy resulting from the change between the two different phases [1,3,8,9]. $T_{m}$, $T_{i}$, and $T_{f}$ are the melting temperature, initial temperature, and final temperature, respectively. The sensible heat storage can be defined by the first and last term of Equation (1), and the latent heat storage is defined by the middle term. The latent heat storage is typically considered a nearly reversible process or in this case, a form of energy storage, and comprises most of the energy stored in the PCM [1]. This process of sensible heat and latent heat changes during phase transitions can also be graphically shown on a T-h diagram (temperature vs. absorbed energy) as shown in Figure 1 [3].

As indicted in Figure 1, the initial slope of the line from the bottom left to the red dashed line represents the solid phase, in which the PCM is absorbing sensible heat (i.e., the temperature of the PCM is noticeably rising). This continues until the temperature of the PCM reaches the melting temperature for a given pressure, which is represented by the red dashed line. As the energy being absorbed is now being used to break the intermolecular forces in the PCM, it will continue to be absorbed and retained/stored and will remain at a constant temperature, while undergoing the phase change from a solid to a liquid. While Figure 1 depicts an ideal scenario, in most practical applications, the temperature varies only slightly during the phase change process, and this variation can typically be reduced through improved mixing. Once the PCM has completely changed phase, only then will the PCM again begin to increase in temperature. Conversely, during cooling, the temperature will remain nearly constant until the phase change has been completed, at which time energy is released and the intermolecular bonds are restored, again causing the temperature to remain constant until the material has returned to its initial state. At this point, the temperature will continue to decrease as it cools, releasing the sensible heat component.

Thorough analyses have been conducted to identify a PCM that meets as many of the ideal properties as possible, resulting in the following list [1]:

1. Optimal melting temperature for specific application,
2. Sizable change in enthalpy between phases,
3. High thermal conductivity,
4. Low volume change between phases,
5. A high density to allow for maximum energy storage,
6. Reversible phase change cycle,
7. Large or small period between complete phase change depending on the application,
8. Non-toxic, non-flammable, non-corrosive,
9. Congruent melting and freezing of the PCM structure.

### 1.2. Nano-Enhanced Phase Change Materials

Phase change materials are typically selected for a specific application based upon the ability to change phase within fairly narrow temperature windows and their large latent heat, number 1 and 2 in the list above, respectively, which makes them well-suited for specific applications. The most significant criteria in the selection of PCMs for a specific application is their thermal conductivity, which governs the charging and discharging times. Even though most of the items in the list can be found in different PCMs, the interaction and related nature of these characteristics makes it very difficult to have all of these ideal characteristics optimized in a single PCM. Though it is difficult to obtain some of these characteristics such as high thermal conductivity, the use of nano-enhanced phase change materials (NEPCM) can, in some cases, help achieve the desired thermophysical properties [3]. 

As NEPCMs are typically homogeneous mixtures of nanoparticles that have been dispersed in more traditional PCMs, they can provide various changes or modifications in the thermophysical properties of the PCM, depending upon the structure and nature of the nanoparticles used. NEPCMs follow the same basic thermal laws as the base PCMs described in Equation (1); however, the values of the specific heat, phase change temperature, charging and discharging times, and the change in enthalpy may all be altered in accordance with the selected nanoparticles, based upon their properties. Aside from these four parameters, the thermophysical property most easily and frequently affected by the introduction of the nanoparticles into a PCM is the effective thermal conductivity [3,10]. 

Applications for NEPCMs are comparable to those of the more traditional PCMs, however, slightly more limited. Not only are they more limited, but they are less likely to be implemented in mainstream applications, due to the remaining uncertainty of how these NEPCMs will perform with various mixtures and various conditions that they are subjected to. NEPCMs could be used for improving building heating and cooling, electronics cooling systems, refrigerators, and other thermal energy storage systems that require rapid thermal cycle times [11]. NEPCMs are currently being used in some applications; however, research is still being conducted in an effort to further optimize them. Common types of nanoparticles dispersed in PCMs include, but are not limited to, carbon nanotubes, carbon nanofibers, and graphite, graphene, metallic, and metal oxide nanoparticles [11,12]. 

### 1.3. Other Methods of Altering Thermophyscial Properties of PCMs

Although the focus of this review is on NEPCMs, there are other methods that can be used to achieve the same objectives as those outlined in the majority of the studies discussed and reviewed here, i.e., to increase thermal conductivity and decrease the charging and discharging time. This section will not cover all methods, but will highlight some of the most common methods currently being used. 

Metal wire mesh is a commonly used mechanism for conducting heat more efficiently through PCMs. To use a wire mesh with a PCM, the PCM is brought to a temperature above the melting point, and then typically held within a mold while wire mesh is immersed into the liquified PCM. Once this has been completed, the PCM is allowed to solidify, thereby trapping the mesh within it. Thus, once the PCM contacts a heat source, the heat will spread through the PCM more evenly and allow for faster charging and discharging times [13,14]. Upon the simple implementation of a raised aluminum mesh into a salt hydrate PCM, a 14% reduction in melting time was observed, and it was numerically determined that a reduction of up to 81% in melting time could be achieved if additional wire meshes were connected thermally in parallel [14]. 

The use of finned walls is also a very common example of how to improve the effective thermal conductivity in order to transfer the heat more rapidly. This form of heat transfer can be seen in many other applications besides from PCMs. Finned structures typically rely on the use of natural convection or forced convection of a fluid on the external side of the apparatus. Convection must be considered in these types of PCMs as well as other heat transfer mechanisms; however, the fins are typically internal appendages that allow for increased distribution of the heat within the PCM [15,16]. Many applications such as solar energy harvesting [16] use horizontal, vertical, or other angled positions. These structures greatly enhance the temperature uniformity and thereby enhance the melting and solidification of the PCM. The number of fins used to heat and cool the PCM can be increased or decreased for specific applications and accommodate the other required constraints, such as overall heat capacity and charging and discharging cycle times. 

In addition to wire mesh and other high conductive materials, heat pipes have also been used to decrease the transient response time between the different phases of a PCM. If applied correctly, heat pipes are very efficient in decreasing the charging and discharging periods, due to their high effective thermal conductivity. For some cases, PCM is used such that an anulus is formed around the condenser end of the heat pipe so that as much surface area of the heat pipe is in contact with the PCM as possible [17]. Heat pipes are also generally used in high-temperature applications, due to their ability to accommodate large heat fluxes at high temperatures. Conversely, depending on the application, PCMs can be used as the cooling mechanism for heat pipes where high levels of heat rejection are required. This can be accomplished through similar methods, i.e., the PCM is wrapped around the evaporator section of the heat pipe to dissipate the thermal energy from the heat pipe [18]. 

Metal foams have also been used as an effective method for increasing thermal conductivity. They are like the wire mesh application, however, in a sense, reversed. The wire mesh is inserted into a PCM in its liquid phase, and then the PCM solidifies around it. Metal foams are porous materials which are used such that the PCM in its liquid form is introduced to this porous media in a confined space so that the PCM fills the pores; once again, the PCM is allowed to solidify and is retained in the metal foam. The effect of this on the effective thermal conductivity of the PCM/foam structure is significant, with the thermal conductivity being governed by the size of the pores in the metal foam, along with the structure and thermophysical characteristics of the metal foam [19]. 

The methods described above are some of the most widely used ways of effectively increasing the thermal conductivity of a PCM. These applications often require the use of a form-stable system, such that either the PCM that has been impregnated into a structure, or a structure that has been implanted into a PCM. The addition of these structures results in an increase in the total volume of the heat storage system, but this is offset by an increase in the overall performance. In addition, it allows the PCM to be less dependent upon the thermal conductivity of the PCM for the distribution of heat throughout the entire structure.

### 1.4. Limiting Factors Affecting NEPCMs

NEPCMs are one potential solution for identifying a material that is capable of both conducting heat, as well as storing thermal energy. Yet, there are limitations that must be addressed to obtain optimal performance from the typical NEPCM. Thermal conductivity is the major limitation yet holds the greatest potential for improvement and hence, is the focus of many previous studies. Having a high thermal conductivity is crucial in effectively transferring the heat throughout the PCM and increasing the thermal conductivity can significantly reduce the time required for charging/discharging (absorbing/releasing energy) of the PCM [20]. Although increasing the thermal conductivity is an important goal/outcome, increases in the thermal conductivity are typically accompanied by decreases in the latent heat, and there are relatively few studies that address both parameters simultaneously [21,22,23,24,25,26,27,28]. Some NEPCMs undergo rapid changes in the structure and thermal performance when subjected to repeated charging/discharging cycles (thermal cycling), and cyclic tests have been conducted to determine the stability and effectiveness of a particular NEPCM [29]. During repeated thermal cycles and the resulting phase change process, the buoyancy forces may result in segregation of the two phases, which can adversely affect the effective thermophysical properties of the NEPCM [30]. A number of investigations have indicated that most organics, such as paraffin and other fatty acids, change very little, even at high cycle counts; some inorganic PCMs cannot withstand as many cycles before their thermophysical properties are susceptible to degradation [29,31]. Thus, for more stable NEPCMs, it may be advisable to use organic PCMs. Segregation also occurs in NEPCMs between the PCM and the nanoparticles when mixed together, causing instability [32]. Thus, stability of NEPCMs when subjected to numerous thermal cycles also presents a potential limitation of NEPCMs.

## 2. Motivation for Review

The motivation for this review was based upon the recent increased interest in applications related to the shipment of cellular-based materials and vaccines, both of which typically require precise temperature control for sustained periods of time. As such, it is limited to NEPCMs that fall within a relatively low temperature range of approximately 0–100 °C. These types of applications may require the transportation of living cellular therapies over moderate to extended periods of time (greater than 4–5 days), usually without the use of or access to external power. The transportation container used must be maintained at constant or near-constant temperatures, since small variations in cell temperatures can be detrimental to the viability of the cells/tissue. As the transportation modalities are typically independent of any external power, PCMs were selected as one of the most viable solutions for maintaining constant temperature within a heavily insulated container. The transportation time of these cells, along with the very small acceptable cell temperature variation, requires that the latent heat of the PCM be maximized, while at the same time minimizing the effective thermal conductivity. This combination of properties provides a sustainable thermal environment, while minimizing the propagation of heat into or out of the internal chamber that hosts the cellular therapies. Due to the sensitivity of these cellular therapies, it is critical that all materials used in the transportation container be as chemically stable and inert as possible to eliminate any potential exposure and contamination of the therapies caused by unintended contact between the PCM and the cellular materials. As a result, a majority of the literature in this review focuses on organics, however, a number of inorganics are included as well due to their potential for high latent heat. To provide sufficient time for the transportation of cellular therapies, it is critical that the nanoparticles within the NEPCM remain suspended over the entire transportation time, including a safety factor for unforeseen delays. This transportation container should ideally be designed for multiple uses, which implies a situation where it will be subjected to numerous thermal cycles. One of the key takeaways from this review will be to determine what research has been conducted on the alteration of thermophysical properties, in particular the increase in latent heat of a PCM and how thermally and physically stable an NEPCM will be after many thermal cycles.

## 3. Review of Current NEPCM Literature

There are several thermal and physical properties that are considered to be important and, in some cases, necessary for PCMs to achieve the requirements previously discussed. Ongoing investigations, both analytical and experimental, have been conducted using various PCMs and nanoparticles in an effort to obtain PCMs with significantly improved thermal properties. For these reasons, the majority of the recent research on NEPCMs has focused on increasing the thermal conductivity. The following subsections present the details of the various PCM studies that have been conducted and the applications on which they are based. 

### 3.1. Commonly Used PCMs and Nanoparticles Used in the Creation of NEPCMs

Examination of current research and experimental investigations indicates that there are several specific types of PCMs that are commonly used for low temperature applications. Table 1 summarizes several of these that are used in NEPCM studies. For more information on each investigation refer to the source cited in the reference section. 

Table 1 summarizes the results from recent analytical and experimental investigations that have been conducted on NEPCMs along with the suggested applications. A more detailed discussion of how the inclusion of the specific nanoparticles, shown in Table 1, may affect the thermophysical properties of their respective PCMs are presented in Section 4 and Section 5 of this review. Paraffin-based materials, water-based solutions, acid-based solutions, and other organic types of PCMs are frequently used for applications in public sectors, due to their chemical composition, which is both safe and, in most cases, chemically inert when used in combination with most nanoparticles.

There are several different nanoparticles and PCM combinations that have been studied and/or used in applications that were not detailed in Table 1 above. However, based upon the information in Table 1 and from the collected literature, some initial inferences can be made. For low temperature NEPCMs, organic PCMs seem to be the most frequently used type of PCM. However, from the examples provided in Table 1, it can be determined that many of the common PCMs that are used in this temperature range are waxes (paraffins) and fatty acids. Additionally, the common types of nanoparticles that can be seen consist of carbon-based materials (carbon black, graphite, graphene), metallic materials, and structured materials (CNTs). Examples of inorganic NEPCMs are also presented in Table 1, which are primarily under consideration for the use of higher latent heat than that of organic NEPCMs for incorporation of the types of applications mentioned above.

### 3.2. Methods Used in the Formation of NEPCMs

During the production of these nanofluids, the PCMs may be subjected to several different processes designed to ensure the proper formation of the NEPCMs. There are several methods in which NEPCMs can be mixed; however, most of these methods can be described as either a one-step or a two-step method. The one-step method refers to the creation of the nanoparticles and mixing of them with the PCMs in a single step. This is often also called production by direct synthesis. These types of processes can be achieved by hot evaporation methods or by chemical production [10,45,46,47]. These processes are often costly; therefore, two-step methods are preferable, until further developments are made in the production processes. The two-step method consists of production of the nanoparticles through physical, thermal, or chemical means, followed by dispersal of the nanoparticles into the PCM. There are several two-step methods that are currently used.

**Figure 2:**
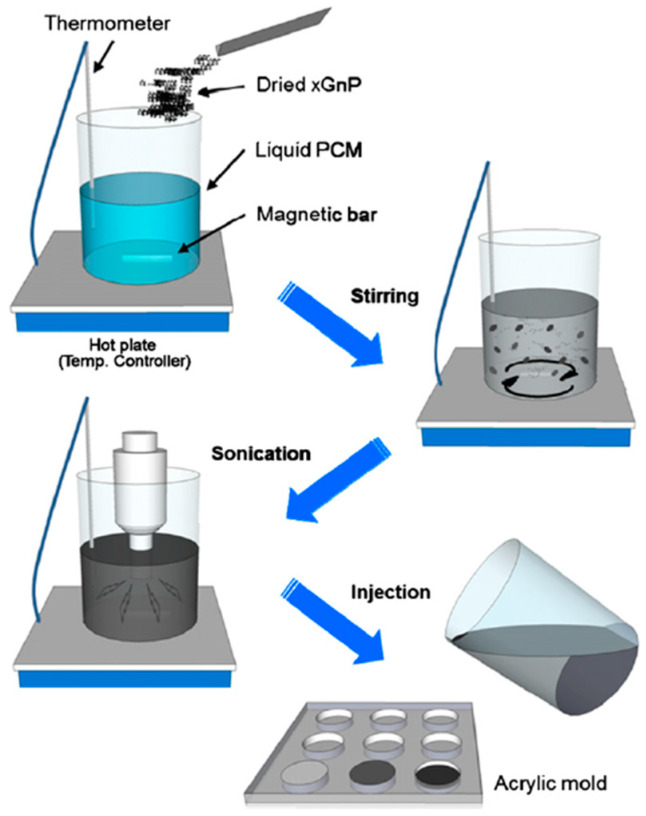
Stirring and Sonication of xGNP nanoparticles into PCM. (Reprinted with permission from ref. [28]. Copyright 2012 Solar Energy Materials and Solar Cells.

Stirring and sonication are both popular methods used to form the NEPCM. In this process, the nanoparticles and PCM are stirred continuously (typically using a magnetic stirrer) for ~30 min and then sonicated for a fixed period. Sonication is the use of vibrational frequencies to continuously agitate the nanoparticles in the PCM to ensure they are thoroughly distributed. An example of sonification for the mixture of nanoparticles and PCM is that of the mixture of xGNPs with hexadecane, octadecane, and paraffin wax, shown in Table 1. A pictorial description of this is illustrated in Figure 2 [28]. 

**Figure 3:**
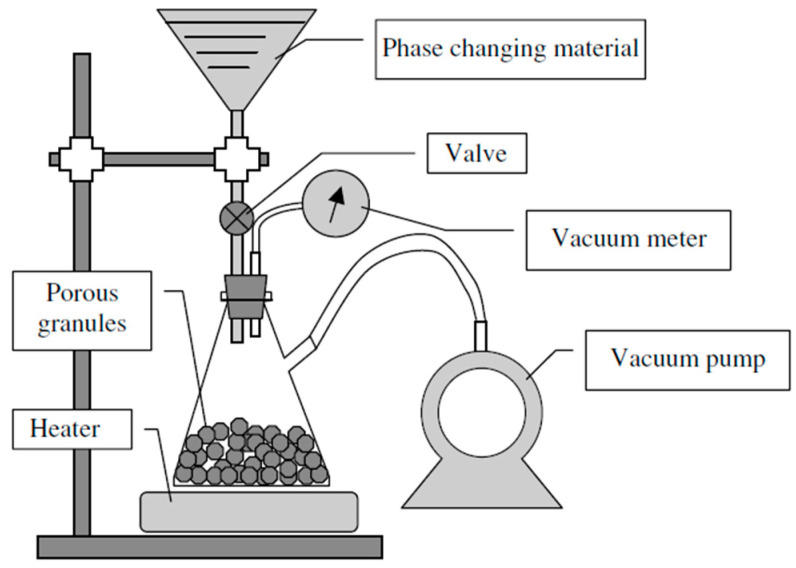
Vacuum impregnation of porous nanoparticles. (Reprinted with permission from ref. [48]. Copyright 2005 Solar Energy).

Similar to sonication is the ultrasonication method, which can negate the stirring method and exposes the mixture to longer periods of agitated vibrations, reaching times that may last in excess of one hour, therefore ensuring optimal dispersion of the nanoparticles, resulting in less error in the measurements of the thermophysical properties [22,24,25,26,27,46]. Another common method used to prepare the PCM and nanoparticle mixture is vacuum impregnation. This method is primarily used for impregnation of PCMs into nanostructures. The nanoparticles are vacuum dried in an oven, then the PCM in a liquid state is slowly introduced into the dried nanostructures. See Figure 3 for a pictorial description) [48]. Refs. [1,24,48] used this form of nanoparticle preparation. Ultrasonication is one of the most frequently used methods for the dispersion of nanoparticles into a phase change material. The process of ultrasonication takes place over a long time; this ensures that the particles are prohibited from uneven heat transfer.

The autoclave, kneader mixing, and varnish layer methods are all other preparation techniques that, while not as common, are viable for certain types of PCMs (see reference [46] for details on these preparation methods).

### 3.3. Characterization Techniques and Measurements of NEPCMs

Nano-enhanced phase change materials are characterized by several specific attributes, and characterizations of these NEPCMs are based upon the thermal conductivity, latent heat, phase change temperature, morphology of the nanoparticles, dispersion quality, viscosity, density, chemical stability, and the structure of the individual nanoparticles [10]. Most commonly, these parameters are measured using equipment specifically designed for nanoparticles. Some parameters, however, can better be determined through theoretical calculations within a reasonably small margin of error, such as thermal conductivity, latent heat, density, and viscosity. Best practices dictate that these values be theoretically determined, then confirmed experimentally, using the necessary equipment and standard test methodologies. 

**Figure 4:**
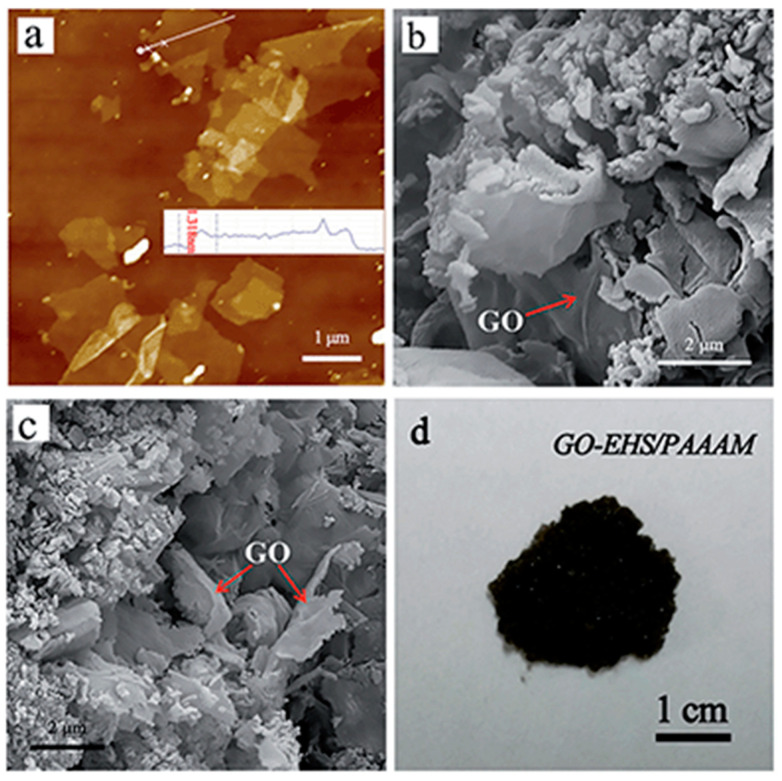
SEM images of graphene oxide nanoparticles used in hydrated salt PCM (**a**) AFM image and height of graphene oxide, (**b**,**c**) SEM images of graphene oxide—EHS/PAAAM, and (**d**) unenhanced image of graphene oxide—EHS/PAAAM mixture. (Reprinted with permission from ref. [38]. Copyright 2013 Royal Society of Chemistry).

**Figure 5:**
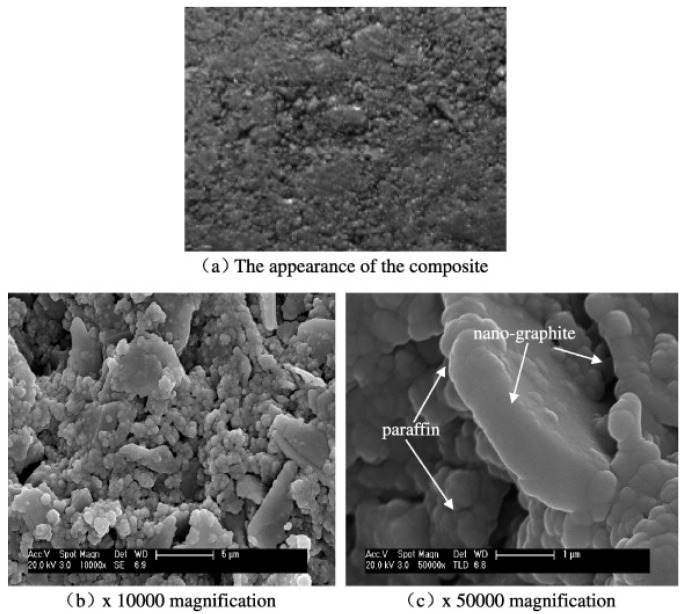
SEM images of paraffin/nano-graphite NEPCM, (**a**) macroscale image of mixture, (**b**) ×10,000 magnified image (**c**) ×50,000 magnified image. (Reprinted with permission from ref. [22]. Copyright 2013 Applied Energy).

**Figure 6:**
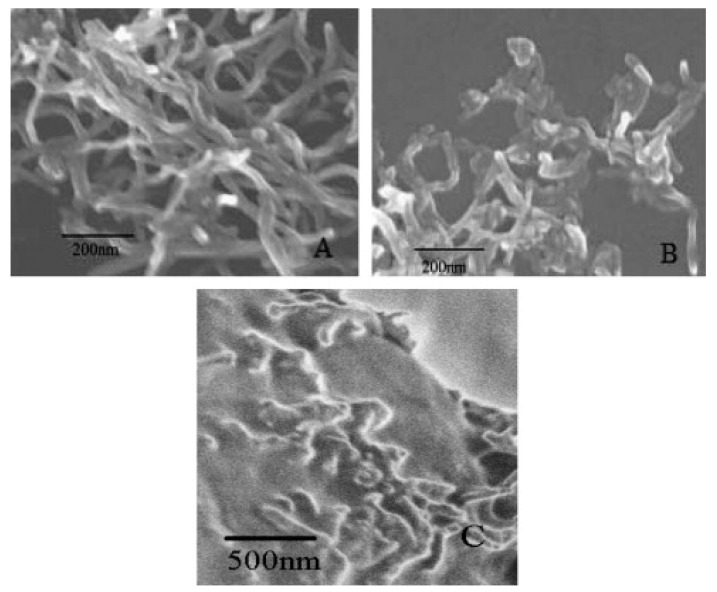
SEM images of palmitic acid/CNT NEPCM: (**A**) pristine CNTs, (**B**) treated CNTs, and (**C**) palmitic acid containing treated CNTs. (Reprinted with permission from ref. [23]. Copyright 2010 Solar Energy).

Scanning electron microscopy (SEM) and transmission electron microscopy (TEM) are typically used for the examination of the dispersal of nanoparticles within the NEPCM and energy dispersive X-ray spectroscopy is used to study the chemical composition of NEPCMs. Differential scanning calorimeters (DSC) are used to determine latent heat and phase change temperatures of PCMs within the compounds. Rotational viscometers are most useful for determining the viscosity of NEPCMs. Thermal conductivity analyzers (TCAs) which come in several forms are typically used for measuring the thermal conductivity of the specific NEPCMs [10,22,27,38,49]. Figure 4, as seen below, is an image of the graphene nanoparticles that have been mixed into a hydrated salt PCM which is described in reference [38]. Figure 5 below represents the example in reference [22] where nano-graphite particles are mixed with paraffin. Figure 5b shows a distinct visual image of the larger graphite platelets and the very small clumps of PCM. Most SEM images of NEPCMs can be used relatively easily to gauge the size of the nanoparticles that are entrained in the PCM. It is also quite easy to tell the difference between the two materials, either by texture or by size. Figure 6 illustrates pristine CNTs, treated CNTs, and the treated CNTs in the palmitic acid PCM in images (a), (b), and (c), respectively [23].

## 4. Results of Previous Studies

Previous investigations are described and summarized in Table 1 and discussed in more detail in Section 3.1 of this review for the more common NEPCMs shown, including the compounds, how they are characterized and what preparation techniques are used to form the NEPCMs. Table 2 below encompasses selected studies summarized in Table 1 and compares the thermal conductivity, latent heat, and phase change temperature between the basic PCM and their NEPCM counterpart. 

### 4.1. Thermophysical Properties

#### 4.1.1. Thermal Conductivity

As discussed previously, the thermal conductivity is the principle governing characteristic for most PCM applications; however, with the inclusion of nanoparticles, the thermal conductivity can be readily improved and typically increases with increases in the nanoparticle mass fraction (wt%), as shown in the examples provided in Table 2. This increase in thermal conductivity can be non-linear and reach a maximum value or begin to behave in a nonlinear fashion with smaller nanoparticle concentrations and then reach a steady linear trend. The following are some examples of these types of relationships between thermal conductivity and nanoparticle concentration. 

**Figure 7:**
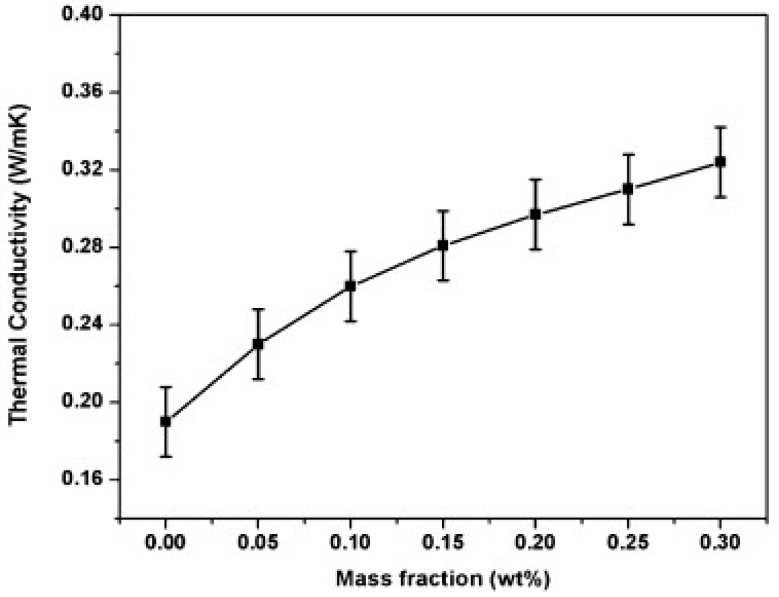
Thermal conductivity to mass fraction relationship for stearic acid-TiO_2_ NEPCM. (Reprinted with permission from ref. [27]. Copyright 2013 Thermochimica Acta).

**Figure 8:**
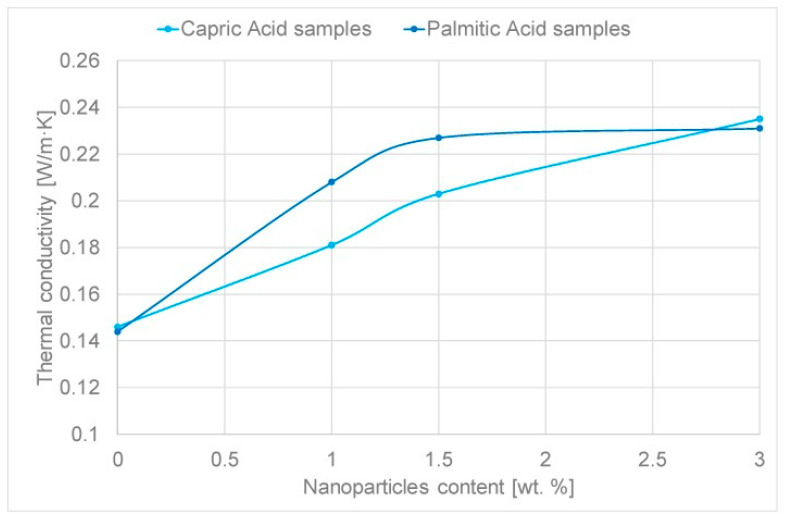
Increase in thermal conductivity of capric and palmitic acid with increase in CuO nanoparticles [50].

**Figure 9:**
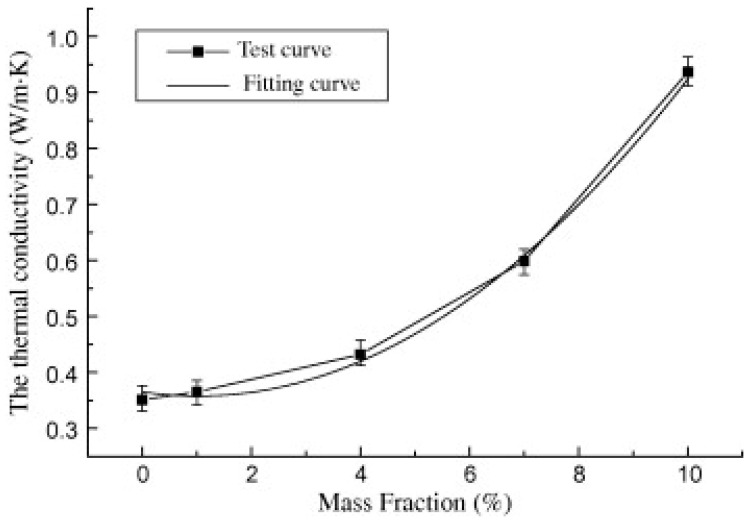
Increase in thermal conductivity of paraffin with increase in graphite nanoparticles. (Reprinted with permission from ref. [22]. Copyright 2013 Applied Energy).

**Figure 10:**
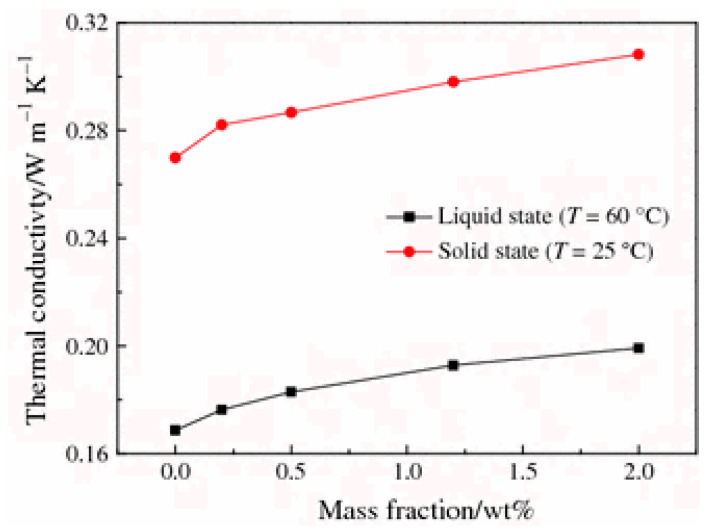
Increase in thermal conductivity of paraffin with increase in copper nanoparticles in the solid and liquid state. (Reprinted with Permission from ref. [51]. Copyright 2011 Journal of Thermal Analysis and Calorimetry).

Steric acid/titanium dioxide exhibits non-linear thermal conductivity trends, as shown in Figure 7 [27]. At values of 0.2 wt% and above the rate of thermal conductivity increase approaches a constant value with the addition of titanium dioxide nanoparticles. Similar non-linear trends are presented in the investigation detailed in reference [50], the addition of copper (II) oxide (CuO) to two organic acids Figure 8 [50]. The thermal conductivity of palmitic acid/CuO NEPCM, shown in Figure 8, reaches a maximum thermal conductivity at a value of approximately 0.23 W/mK when the nanoparticle mass fraction reaches roughly 1.5%. This differs from the capric acid trend from the same figure and that of the steric acid in Figure 7. The investigation performed on paraffin and nano-graphite NEPCM, seen in Table 2, also exhibits this trend. Figure 9 [22] illustrates the relationship between thermal conductivity and the mass fraction of nanoparticles. As was the case for Figure 7, Figure 8 and Figure 9 also portrays a non-linear trend, similar to a quadratic relation [22]. NEPCM exhibited similar trends for thermal conductivity in the liquid or solid state. Figure 10 illustrates this relationship for paraffin PCM with Copper nanoparticles for a range of 0 to 2.0% mass fraction [51].

Based upon this information, it can be inferred that the thermal conductivity of NEPCMs can increase to an equilibrium mass fraction of nanoparticles and then remain constant and independent of the mass fraction, or reach a steady rate of increase in the thermal conductivity with an increase in the mass fraction. The first case eliminates the need for continued increases in the mass fraction beyond the equalization value if the NEPCM follows this pattern; however, when the latter is true, an optimization mass fraction of nanoparticles must be determined to ensure optimal performance of the NEPCM. A balance between optimal thermal conductivity with minimal reductions in latent heat is preferred. These conclusions are not definitive in studies that have a limited number of data points but appear to be quite common and consistent.

#### 4.1.2. Charging and Discharging Time 

**Figure 11:**
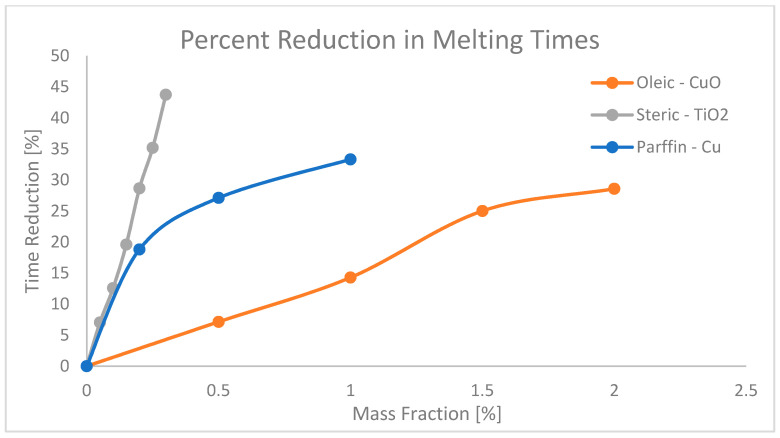
Percent reductions in melting times of paraffin, oleic and steric acids from references [27,51,52].

**Figure 12:**
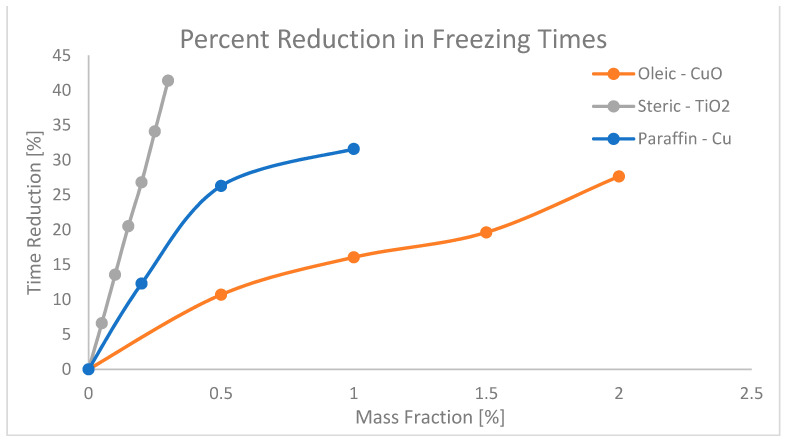
Percent reductions in freezing times of paraffin, oleic and steric acids from references [27,51,52].

Increasing the thermal conductivity in NEPCMs allows for the absorption and release (charge and discharge) of thermal energy rapidly. This in turn allows the entire process of charging and discharging energy to occur over shorter time durations, making these NEPCMs particularly useful for applications where rapid response times and short time constants are desirable. Experiments have been conducted to determine how charging and discharging times are affected with the increase in nanoparticle concentration. Wu et al. [51] studied the effects of copper nanoparticles added to a paraffin PCM in increments of nanoparticle mass fraction, to determine the charging and discharging time. As a baseline, pure paraffin was recorded to melt at ~48 min, whereas when 0.2%, 0.5%, and 1.0% mass fraction of nanoparticles were added to the paraffin, the melting times decreased to 39 min, 35 min, and 32 min respectively. This resulted in a maximum decrease in melting time of the NEPCM by 33.3%. The reverse process was then conducted to record the freezing time improvements. For the baseline, 57 min was recorded for pure paraffin, then following the same process for the mass fractions listed above, times were recorded as 50 min, 42 min, and 39 min, respectively. This resulted in a maximum decrease in freezing time of 31.6% [51]. Another investigation performed on the combination of oleic acid and copper-oxide (CuO) nanoparticles was performed by Harikrishnan [52]. This investigation did not include a base line for time, but rather described the relative percent increase in melting and freezing time frames. When tested with 0.5%, 1.0%, 1.5%, and 2.0% mass fractions, the melting times decreased by 7.14%, 14.28%, 25%, and 28.57%, respectively, and the freezing times decreased by 10.71%, 16.07%, 19.64%, and 27.67%, respectively [52]. Additionally, in a study by Harikrishnan [27] of steric acid, TiO_2_ NEPCM indicates that the melting time of basic steric acid is 16.9 min, and the freezing time is 25.2 min. Concentrations of 0.05%, 0.1%, 0.15%, 0.2%, 0.25%, 0.3% mass fraction of TiO_2_ nanoparticles were tested and resulted in a reduction in the melting times of 7.03%, 12.56%, 19.59% 28.64%, 35.17%, and 43.72%, respectively, accompanied by freezing time reductions of 6.62%, 13.57%, 20.53%, 26.82%, 34.11%, and 41.39%. The reduction percentages described above have been collected and plotted for visual representation of the effect of nanoparticle mass fraction on the melting and freezing time in Figure 11 and Figure 12, respectively. 

From this analysis, the results for most NEPCMs that contain nanoparticles with a higher thermal conductivity than the basic PCM indicates that the incorporation of nanoparticles that have a higher thermal conductivity than the PCM, when dispersed into the PCM, results in an overall increase in the thermal conductivity. Thus, the higher the mass fraction of nanoparticles, the more conductive the NEPCM will be. If a material’s thermal conductivity is increased, heat will transfer more easily through the material, making it easier for NEPCMs to change state more uniformly when compared to the base forms.

#### 4.1.3. Latent Heat 

**Figure 13:**
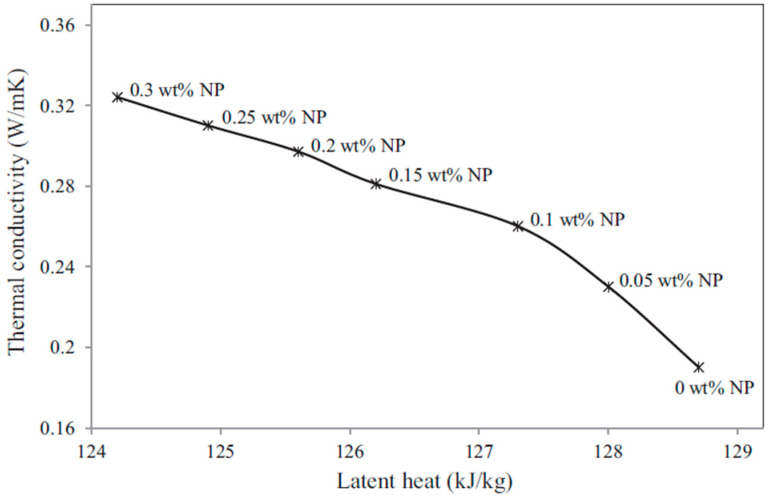
Thermal conductivity, latent heat, and nanoparticle wt% of stearic acid. (Reprinted with permission from ref. [27]. Copyright 2013 Thermochimica Acta).

As mentioned above, if an excess of nanoparticles is used, then significant decreases in latent heat can occur, causing the NEPCM to decrease in effectiveness. The addition of most nanoparticles to a PCM will decrease the latent heat value, thereby reducing the amount of thermal energy that the NEPCM can absorb and release [1,2,3,10,11,12,13,14,15,16,17,18,19,20,21,22,23,24,25,26,27,28,29,30,31,32,33,34,35,36,37,38,39,40,41,42,43,44,45,46,49,50]. Even though thermal conductivity is a major focus of NEPCMs, for most applications, the latent heat values are the most important thermophysical property of a PCM. The trend of decreasing latent heat with increasing nanoparticle mass fraction can be seen in most of the references listed in Table 2. Figure 13 illustrates the relationship between the mass fraction of the nanoparticles, the thermal conductivity, and the latent heat, as presented in reference [27]. This information is also referenced in Table 2. 

**Figure 14:**
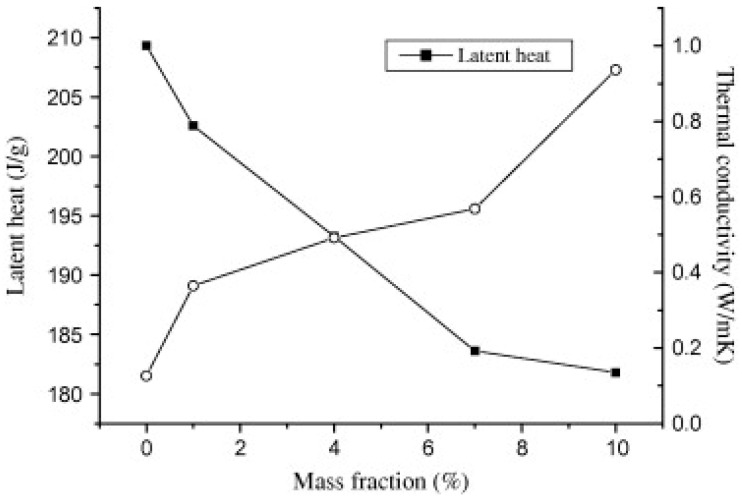
Latent heat vs. thermal conductivity vs. mass fraction of paraffin/nano-graphite NEPCM. (Reprinted with permission from ref. [22]. Copyright 2013 Applied Energy).

In the example of the paraffin PCM and the graphite nanoparticles, another prime example of the effect that mass fraction has on thermal conductivity and latent heat is apparent [22]. As the mass fraction increases the thermal conductivity increases as well, and the latent heat decreases; see Figure 14. This has an overall negative effect since latent heat is often important and a deciding factor in the selection process, ensuring that the thermal system has the thermal capacity for a specific application. In this example as well as others, there is no generalized method for determining the optimum balance between thermal conductivity and latent heat. This is typically solely dependent upon the application of the NEPCM. If the thermal system requires an NEPCM that can absorb and release energy quickly, then a higher mass fraction of nanoparticles would be the better choice, but if a higher energy storage is required, then the lowest possible mass fraction without jeopardizing the thermal system would be preferable. 

There are several reasons why general trends of increasing thermal conductivity and decreasing latent heat occurs with increases in the mass fraction of nanoparticles. If the specific heat of the nanoparticles is much less than that of the PCM, it may decrease the ability to absorb heat. Another possible cause for the decrease in latent heat is the disruption of the molecular forces. If these forces and bonds are weakened by nanoparticles, it no longer requires as much energy to break them, thereby absorbing less energy [3,20].

The aggregation of nanoparticles can significantly affect both the thermal conductivity and the heat storage of NEPCMs. There are optimal-sized aggregates that result in the higher thermal conductivity and heat capacity. These optimal sizes are typically on the very small scale, whereas aggregates that are on the micrometer scale typically result in a decrease in these properties. Additionally, the converse of the first statement could be true, such that increases in total heat storage could also be connected to the specific heat of the nanoparticle. The literature indicates that with the addition of nanoparticles, the specific heat of the mixture will change depending upon whether the specific heat of the nanoparticles is higher or lower than that of the base PCM. Little analysis of the comparison between the sensible heat and latent heat of the NEPCMs was found; however, it is reasonable to assume that if the same mass of a particular PCM is compared to the nanoparticles, it may be very possible that the sensible heat stored in these nanoparticles is greater than that of the PCM. As a result, the overall media would then have significantly different thermal and physical properties. At this point, the issue is whether the nanoparticle sensible heat storage capability outweighs that of the latent heat of the PCM when mixed. The majority of the literature reviewed has not performed this analysis; as a result, this is an area that may merit some additional analysis [34,53].

#### 4.1.4. Phase Change Temperature

**Figure 15:**
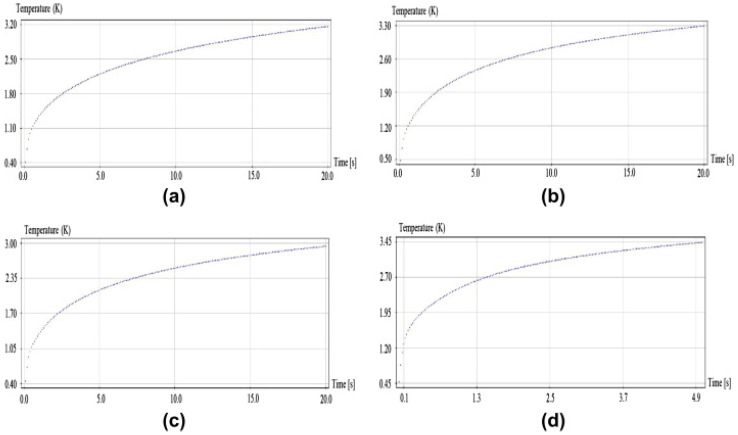
Paraffin/nano-graphite phase change temperature. (Reprinted with permission from ref. [22]. Copyright 2013 Applied Energy).

No specific trends in the phase change temperature of these material combinations are apparent from this review. Phase change temperatures may increase, decrease, or in some cases change very little with increases in the mass fraction of nanoparticles in the PCM. There are several examples in Table 2 that illustrate the variations in the phase change temperatures with respect to nanoparticle mass fraction. From a reliability perspective, it is best if there is little to no variation in the phase change temperature, since if it is subjected to numerous thermal cycles, the phase change temperatures may begin to vary, and hence, the performance of the NEPCM may decay over time or repeated cycling [27]. Once again, a good example of these trends is illustrated in paraffin PCMs with nano-graphite nanoparticles. The base phase change temperature for this NEPCM is 28.81 °C. The values for the added mass fractions can be seen in Table 2 and range from 1% mass fraction to 10% mass fraction. As shown in Table 2, along with the trends seen below in Figure 15, the phase change temperature slightly decreases from that of the original PCM, yet there is not a significant difference. This is an example of an optimal NEPCM that indicates little to no change in phase change temperature. If the phase change temperature were to change drastically with the addition of nanoparticles, the possibility of a reduction in efficiency due to failure to reach the desired temperature exists. Figure 15 is also a good example of how the addition of nanoparticles can affect the charging time of an NEPCM. It should be noted that if Figure 15 is viewed from (a) to (d) in increasing mass fraction, the temperature reaches the desired phase change temperature more rapidly.

**Figure 16:**
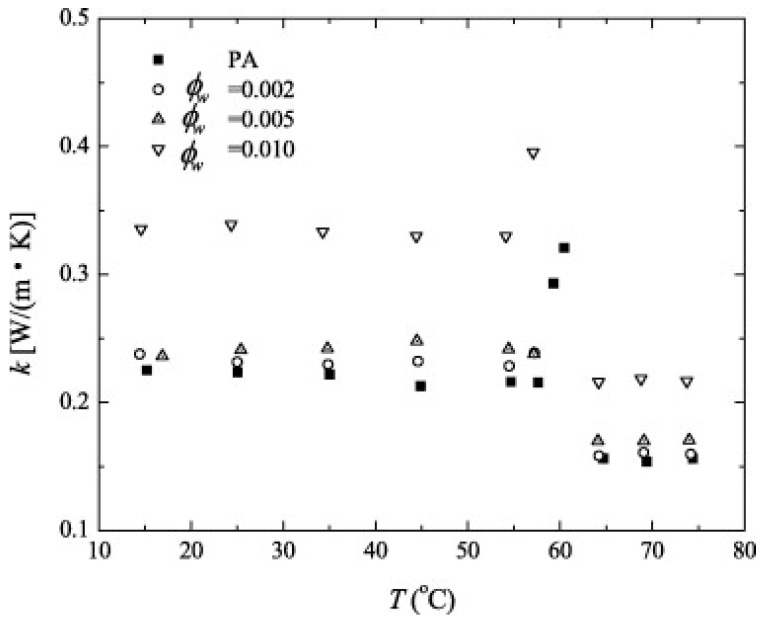
Temperature, thermal conductivity, and mass fraction reaction for palmitic acid/CNT NEPCM. (Reprinted with permission from ref. [23]. Copyright 2010 Solar Energy).

Palmitic acid as the PCM with CNT as the nanoparticle (see reference [23]) also portrays an optimal NEPCM. It can be seen from Table 2 that the phase change temperature varies just over one degree Celsius, as shown in Figure 16. The horizontal variation between the point markers is the difference between the phase change temperature at either 0.2%, 0.5%, or 1.0% mass fraction. The variation is very minute. Additionally, from this figure, it can be visualized how the thermal conductivity is dependent upon the phase change temperature. There is a slight downward trend from phase temperatures of 15 to 55 °C. This is also a graphical representation of how the thermal conductivity can dramatically change when the NEPCM completely changes phase, which is seen by the sudden drop in thermal conductivity after ~60 °C. Variations in the phase change temperature for studies performed in references [27,28] are illustrated in Table 2. If the study performed in reference [26] (Table 2) with a PCM of n-octadecane and nanoparticles of alumina is analyzed, several trends become apparent. Most importantly is that even though the results indicate that there is little variation in the phase change temperature, there is with large increases in mass fraction from 0 to 10%; the thermal conductivity barely increases at the cost of large reductions in the latent heat. From this, one can conclude that this NEPCM does not perform very well since a significant amount of energy storage capacity must be lost for a minimal return in thermal conductivity. 

### 4.2. Stability of NEPCMs

Depending upon the application for which a specific NEPCM is being used, the relationship between viscosity and sedimentation could be either beneficial or detrimental. Viscosity is the principal parameter that governs the level of frictional resistance a particular fluid presents to the flow, while sedimentation describes the tendency for one material, in this case, the individual nanoparticles, to settle within the PCM or base fluid, which results in the segregation of the nanoparticles and the base fluid. In virtually all cases, the addition of nanoparticles to a liquid increases the viscosity, resulting in decreased motion of the nanoparticles. However, this increase may be offset by the increase in other parameters such as thermal conductivity, etc. It is quite common for the nanoparticles and the PCMs to be of different densities, causing the natural separation or settling of the two over time. The viscosity of the NEPCM is also dependent on the temperature, thus one of the main objectives can be maintained, keeping a relatively constant temperature window, the viscosity will be less dependent on the temperature.

**Figure 17:**
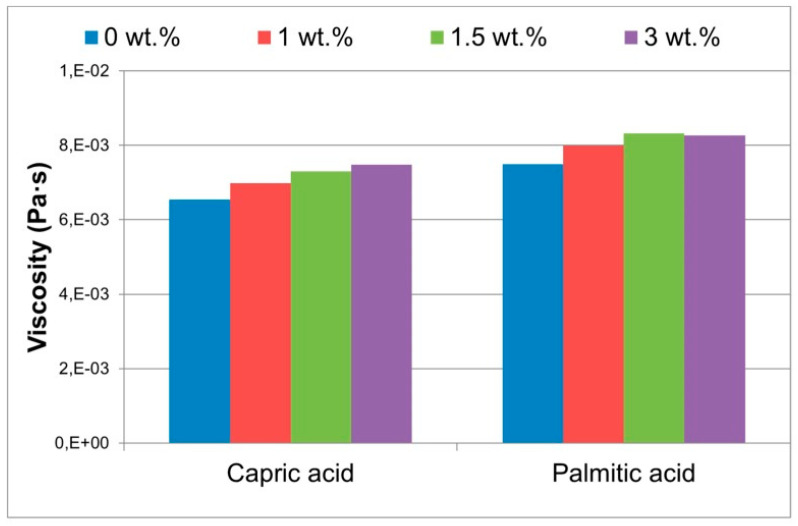
Effect of nanoparticle mass fraction on viscosity of the capric acid and palmitic acid NEPCM [50].

The sedimentation rate of the nanoparticles, whether they are more or less dense than the PCM, is dependent upon both this density difference and the viscosity. The more viscous a liquid, the slower the sedimentation rate [3,50]. Figure 17 illustrates the increase in the viscosity of capric acid and palmitic acid with an increase in the copper (II) oxide nanoparticle mass fraction [50]. Closer examination of these data indicates that in this example, the mass fraction of 3% appears to be the maximum viscosity and is very close for both acids. As such, it would result in the slowest sedimentation rate. However, this higher mass fraction would also result in a lower latent heat and a corresponding reduction in the amount per unit volume of heat that can be absorbed. 

When nanoparticles are introduced into a PCM, especially for those that are normally in the liquid phase, the nanomaterials may form aggregates or clump together [54]. Several steps can be taken to reduce this tendency, and thereby decrease the sedimentation rate. To determine the applicability of a specific NEPCM and ensure that it is sufficiently stable to be used in long-term applications, it is necessary to measure the sedimentation rate. First, as discussed previously, ultrasonication can be used to disperse the nanoparticles [55,56]. Second, surfactants or other additives can be used to increase the surface tension and thereby increase the forces that govern the suspension of the nanoparticles. Third, a compound can be applied on the surface of the individual nanoparticles, which will increase the zeta potential [57]. The zeta potential represents a measure of the electrostatic charge of the nanoparticles. The higher the value of the zeta potential the more electrostatic repulsion between the particles, thus making the NEPCM a more stable mixture [55,56,58]. Finally, increasing the repulsive charge of the particles through variations in the pH resulting from the addition of a surface control chemical may be used to create a high surface charge, which in turn causes repulsive forces in the NEPCM [54]. Although these are the primary mechanisms, reference [54] describes several other methods that can be used to both modify and measure the zeta potential of the NEPCM. 

The first and second methods tend to be the most commonly used forms of insuring that the dispersion of the nanoparticles is adequate. However, it has been shown that the addition of chemical surfactants can have many negative outcomes. As a result, care should be taken as to the amount of surfactant that is added to the NEPCM, as too little will not adequately compensate for the aggregation of the nanoparticles [54]. Conversely, the addition of too much of a surfactant can result in contamination of the NEPCM. Finally, the use of surfactants can produce heat foams during heating and cooling processes [47,59]. 

Ultrasonication is a more readily available method and mitigates the potential of chemical instability. However, for some NEPCMs, there are certain optimized durations that an NEPCM should not be subjected to, as further ultrasonication can have the opposite effect and cause agglomeration of nanoparticles [54]. It has also been observed from the various preparation methods used in the literature reviewed here, that surfactants and ultrasonication can be used together. The goal of combining these processes should be to reach the highest possible dispersion. This is often accomplished by the combination of two of these methods for increasing the zeta potential. For long-term use, the introduction of other chemicals to the NEPCM is recommended.

Sami and Etesami conducted an experiment using TiO_2_ nanoparticles and paraffin as the PCM. This study was conducted to determine the effectiveness of using a surfactant to help stabilize the nanoparticles in the NEPCM and decrease the sedimentation rate. Sodium stearoyl lactylate (SSL) was used as the surfactant. For this investigation, two samples were tested at a mass fraction of 3% of TiO_2_. One of the samples contained SSL while the other did not. Below, Table 3 summarizes data collected from experiments conducted in [60]. 

The degradation of the phase change temperature and latent heat were greater in the sample without SSL; however, the thermal conductivity had greater degradation in the sample with SSL. While this appears to be promising, these represent only a limited number of cycles. To test the true long-term stability, the samples would need to be subjected to a greater number of thermal cycles.

**Figure 18:**
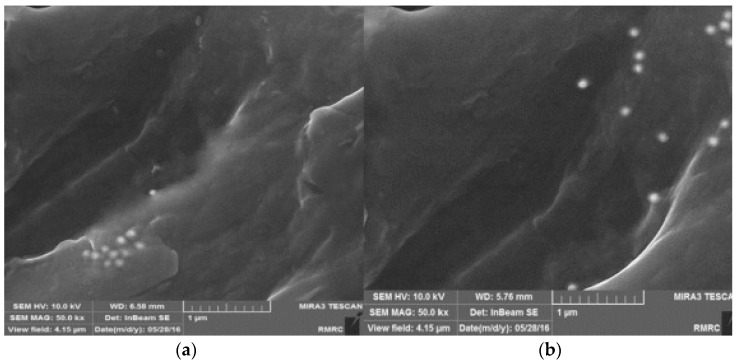
Aggregation of TiO_2_ nanoparticles in paraffin PCM, (**a**) without SSL, and (**b**) with SSL. (Reprinted with permission from ref. [60]. Copyright 2017 Applied Thermal Engineering).

Figure 18 [60] illustrates an SEM image of the two TiO_2_ samples after the 80 thermal cycles had taken place. The image on the left is without SSL and the image on the right is with SSL. Thus, it is easy to conclude that using SSL is more effective at keeping the NEPCM stable than just the NEPCM by itself. Another investigation conducted by Masoumi et al. also used TiO_2_ nanoparticles for dispersion in steric acid [61]. This experiment utilized the sedimentation balance method in which a tray is immersed into the NEPCM and the suspension fraction is calculated by the weight, read from the balance. The suspension fraction is a simple calculation of Fs = (W_0_ − W)/W_0_, for which W_0_ is the total mass of the nanoparticles [58]. For the steric acid-TiO_2_ investigation, four mass fractions were tested at 1%, 2%, 3%, 5%, for the suspension fractions of 0.122%, 0.366%, 0.554%, and 0.878% respectively. This investigation demonstrated that the lower concentration of nanoparticles, resulting from the addition of a surfactant, resulted in the least amount of sedimentation. These values were recorded through the subjection of 250 thermal cycles to prove longevity [61].

## 5. Discussion of Potential Future Developments

Recent investigations of NEPCMs have resulted in a number of significant discoveries, making them of increasing interest, especially in the field of renewable energy. However, there are still a number of issues that require additional investigation. Following is a discussion of future work that is recommended to better understand the impact of the various parameters that govern the behavior of the NEPCMs.

### 5.1. Increase in the Latent Heat

While significant progress has been made in the enhancement of thermal conductivity and the rapid heating and cooling of NEPCMs, most of this progress has focused on the need for higher thermal conductivities and reductions in the time required for charging and discharging. Although these are important parameters, there are some applications where increasing the thermal conductivity is not a primary concern. In fact, in many applications, it is more important that the focus be placed on identifying additional methods for increasing the latent heat of the PCM. For scenarios where the PCM is used in cold chain or ambient atmospheric conditions, insulation is quite important. Rather than focusing on the speed at which the melting and solidification of the PCM processes occur, it may be advantageous to slow the process such that it occurs over an extended period, resulting in a more uniform temperature profile. The use of a lower thermally conductive PCM results in a system that is more thermally resistant, thus makes it more challenging for heat to propagate through the system. One such application is the case of the shipment of cell therapies, where it is necessary for stem cells to be transported for extended periods of time at a constant temperature. One method by which this can be accomplished is to heavily insulate the container used for the transportation of the cells. In addition, the overall thermal resistance of the system can be enhanced by using a PCM that not only has larger latent heat but also contributes to the thermal insulation of the container as well. Thus, a numerical study is currently underway to determine if having a more uniform melting or solidifying temperature profile is more advantageous than having a PCM with high thermal resistance. To this end, the experimental studies conducted on PCMs to determine a combination of nanoparticle and PCM to increase the overall latent heat of the NEPCM and a low thermal conductivity are very limited in number.

**Figure 19:**
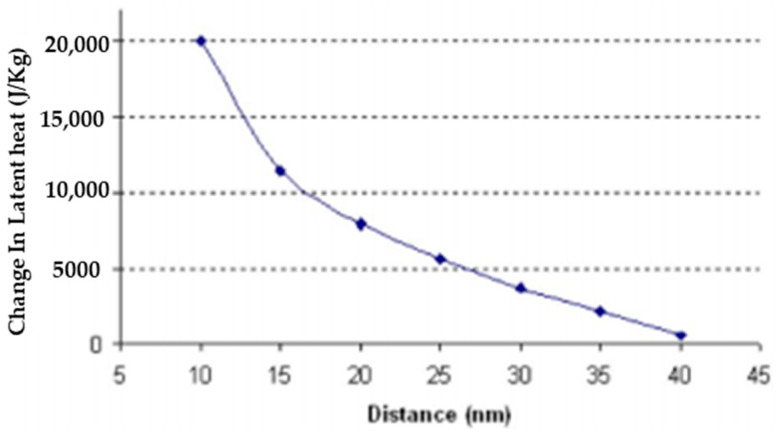
Relationship between latent heat transfer of shell wax/CNT NEPCM with distance between the CNT nanoparticles and PCM molecules. (Reprinted with Permission from ref. [33]. Copyright 2008 Journal of Applied Physics).

In a related investigation, three types of carbon nanoparticles were introduced into paraffin wax. These nanoparticles consisted of single wall carbon nanotubes (SWCNT), multi-walled carbon nanotubes (MWCNT), and carbon nanofibers (CNF). The argument made was that if carbon nanoparticles can form a stronger intermolecular bond with the PCM particles than the PCM particles have with each other, it would require more energy to break the bonds, thus increasing the latent heat. SWCNTs were shown to perform best with increasing intermolecular forces resulting in a maximum increase in the latent heat of 12.98% over that of pure paraffin [33]. If other nanoparticles can be found that can form a stronger intermolecular bond with paraffin waxes or other PCMs, it may be possible to increase the latent heat even further. These claims were supported by the numerical simulation of how the latent heat transfer was affected by the distance between the CNT and the PCM molecule. The results indicated that as the distance between the PCM molecule and the CNT increased, the latent heat would decrease due to the weakening of the intermolecular bond. Figure 19 illustrates this relationship [33]. Although this investigation was able to support its findings, there was an assumption made that could jeopardize the NEPCM if it were to ever be used in long-term applications. To perform the numerical calculations, it was assumed that the arrangement of the nanoparticles dispersed throughout the PCM were of a square lattice formation. This may be an acceptable assumption to make when the first test is being conducted; however, if this NEPCM were to be subjected to long-term thermal cycling, it may likely experience sedimentation effects as described previously. With the formation of aggregates or clusters of nanoparticles, the assumption of uniformity in the substance will likely no longer apply. Therefore, it would be recommended that additional experiments be carried out on a CNT NEPCM similar to this to test the effectiveness of this solution after being subjected to many thermal cycles.

An investigation based on the combination of two types of commercial grade paraffin wax and two different nanoparticles was conducted. Two variations of paraffin wax (RT20 and RT25) were used in an experimental test in which aluminum oxide (Al_2_O_3_) and carbon black (CB) nanoparticles were combined with a 1.0% mass fraction of nanoparticles for both combinations. Four samples were fabricated; i.e., each of the two paraffins were combined with each of the nanoparticle types. The base thermal conductivity of RT20 and RT25 are 0.254 and 0.260 W/mK, respectively. When the nanoparticles were mixed in the RT20 PCM, the thermal conductivities for Al_2_O_3_ and CB were 0.234 and 0.344 W/mK, respectively. For RT25 NEPCMs, the thermal conductivities of Al_2_O_3_ and CB were 0.242 and 0.323 W/mK [35]. This is very unusual, as typically nanoparticles increase the thermal conductivity; however, the Al_2_O_3_, when added, decreased the thermal conductivity of the nanosuspension. Furthermore, the specific heat was recorded for each of the four samples over a temperature range of 10 °C to 67 °C. The recorded values of specific heat were used to create a best fit curve and this function was then integrated to determine the latent heat of the NEPCMs. The base latent heats of RT20 and RT25 are 117.8 J/g and 133.5 J/g, respectively. When the nanoparticles were mixed in the RT20 PCM the latent heat values for Al_2_O_3_ and CB were 130.5 and 121.8 J/g. For RT25 NEPCMs, the latent heat values for Al_2_O_3_ and CB were 134.2 and 117.9 J/g. For the RT20/Al_2_O_3_ this is nearly an 11% increase in latent heat, and 3% for CB. However, for the RT25/Al_2_O_3_, this is only a 1% increase in latent heat, but 12% for CB [35]. From these results, the differences in the thermal conductivity and latent heat for either nanoparticle was strictly dependent upon the base PCM. However, the trends of RT20 and RT25 are not consistent. The Al_2_O_3_ appears to decrease the thermal conductivity and increase the latent heat. Thus, it should be noted that the increase in the nanoparticle density increases the phase change material specific heat [35]. 

### 5.2. Effects of Sedimentation 

As discussed previously, sedimentation is the process whereby the PCM and the nanoparticles separate and stratify in the compound. This may not be an important factor if the liquid has a low enough viscosity and is in continuous motion, as this results in a continuous disturbance of the nanoparticles, resulting in a well-mixed nanofluid. However, if an NEPCM is used for an application in which the fluid motion is stagnant when in the liquid phase, sedimentation could result in significant changes in the thermophysical properties of the NEPCM. As described in reference [51], the sedimentation velocity (i.e., how fast the nanoparticles congregate to the top or bottom of the containment vessel) can be calculated using Equation (2) below:

(2) $$V=\frac{2\times r^{2}\times \left(\rho_{np}-\rho_{pcm}\right)\times g}{9\times \eta }$$

where *V* is the sedimentation velocity; *r* is the nanoparticle radius; *ρ_np_* and *ρ_pcm_* are the densities of the nanoparticles and phase change material, respectively; η is the dynamic viscosity of the mixture; and *g* is the local gravitational acceleration. It is apparent from this equation that to ensure that the sedimentation velocity is very slow, the densities of the two materials must be approximately the same. In this investigation, capric acid and palmitic acid with CuO nanoparticles were evaluated as various nanoparticle mass fractions. The slowest velocity was 2.89 × 10^−9^ meters per second, which was judged to be too slow to account for any difference, i.e., the actual settling velocity units most appropriate would be approximately 9.1 centimeters/calendar year. However, while this is not rapid by any means, it could create a problem in long-term stable applications where no other mixing occurs. There is little information in the literature related to this situation, which makes it an interesting topic for future research; i.e., how would one facilitate the mixing of nanoparticles to avoid the separation of the PCM and nanoparticles to ensure constant and uniform thermophysical properties? Are there other methods aside from the addition of other chemicals? Could a highly viscous PCM be used instead, such that phase change can still occur, but in the liquid form it is still too viscous for the segregation of aggregation of the nanoparticles?

## 6. Conclusions

Several novel approaches for enhancing thermophysical properties of PCMs have been developed and utilized. However, the use of NEPCMs provides numerous ways in which thermal energy storage can be applied. As demonstrated by this review, several trends and/or tendencies are apparent. It is important to note that the majority of these studies are primarily focused on the enhancement of thermal conductivity and reducing the thermal cycling time (charging and discharging times. These can be summarized as follows:

- Organic, inorganic, and a mixture of the two can successfully be used as NEPCMs; however, for the limited range of low-temperature PCM of this review, organics seem to be the most common. Organic PCMs are more commonly used and largely effective in applications where chemical stability and a low temperature environment is important.
- For organic PCMs, metallic nanoparticles and carbon-based (graphite, graphene) nanoparticles seem to be most effective in changing thermal conductivity and decreasing charging and discharging times; however, these tend to decrease the latent heat significantly. CNTs, however, seem to lead to a smaller increase in thermal conductivity and charging discharging time than metallics or carbon-based nanoparticles.
- As noted previously, the trends mentioned in the previous bullet are absolute. For instance, the CNTs and the aluminum oxide discussed in Section 5.1 lead to the increase in latent heat when mixed with paraffin. This leads to the conclusion that most of the thermal effects caused by nanoparticles are case-specific, thus making it difficult to predict the outcomes of PCM and nanoparticle combinations unless they are very similar to those that have already been studied.
- The stability of nanoparticles in a PCM plays a significant role in how effective an NEPCM is. If aggregation occurs, it is likely that the NEPCM will experience rapid degradation of thermal properties, such as thermal conductivity, phase change temperature, and latent heat. If sedimentation rates are rapid, then this results in lower thermal cycle tolerance, which prevents the NEPCM from being eligible for long-term applications and studies.
- A general trend is apparent in which an increase in mass fraction of the nanoparticles, results in a corresponding increase in the thermal conductivity and sedimentation rate and a decrease in latent heat; only slight changes in the phase change temperature have been observed, and these were primarily case-specific.
- It is recommended that additional studies be conducted with the goal of determining the types of nanoparticle and PCM combinations that result in significant increases in latent heat, so that more thermal energy can be stored in the same volumetric space. It is also recommended that more in-depth research be conducted on the most effective ways to suspend nanoparticles in the NEPCM mixture to greatly reduce the sedimentation rates. This could be possible through the further development of intermolecular bonds or the possible creation of a new PCM that maintains high viscosity.

## Figures and Tables

**Table 1:** Samples of commonly used PCMs and nanoparticles.

Ref.	PCM	Nanoparticles	Goal of Literature	Potential Applications
[22]	Paraffin wax	Nano-graphite	Increase thermal conductivity.	Generalized thermal energy storage.
[23]	Palmitic acid	Carbon nanotubes (CNT)	Increase thermal conductivity.	Generalized thermal energy storage.
[24]	n-octadecane	TiO_2_ (Titanium Dioxide)	Increase the thermal conductivity and study the effects on liquid phase viscosity/density of particles. Affects with variation of temperatures.	Generalized thermal energy storage.
[25]	Water based Nanofluid	MWCNTs	Increase the thermal conductivity and minimize solidification times.	Potential use for rapid cooling of low temperature thermal energy storage.
[26]	n-octadecane	Alumina (Al_2_O_3_)	Investigations of affected thermal properties: latent heat, density, viscosity, and thermal conductivity.	Useful for thermal energy storage except for those dominated by natural convection heat transfer methods.
[27]	Stearic acid	TiO_2_ (Titanium Dioxide)	Increase in phase change temperature, decrease in latent heat, and increase in thermal conductivity with the increase in wt% of nanoparticles of the compound.	Used for the applications of solar heating systems.
[28]	Hexadecane Octadecane Paraffin Wax	Graphite nanoplatelets	Thermal conductivity analysis of thermal properties.	Testing for use in floor heating applications.
[33]	Shell Wax	CNT	Increase latent heat.	Generalized thermal energy storage.
[34]	Paraffin (RT20) Paraffin (RT25)	Aluminum Oxide Carbon Black	Increase latent heat. Increase thermal conductivity.	TES and passive cooling systems
[35]	Polyethylene Glycol (PEG)	Carbon Black	Analysis of thermal properties.	Generalized thermal energy storage.
[36]	1-Dodecanol	Graphite Nanosheets (GNSs)	Thermal conductivity, latent heat, specific heat and viscosity analysis.	Thermal energy storage.
[37]	Tetradecanoyl	Copper Nanowires (CuNWs)	Analysis of general thermal properties.	Solar energy storage.
[38]	Sodium Carbonate Decahydrate/PAAAM Sodium hydrogen phosphate dodecahydrate/PAAAM	Graphene oxide	Enhance thermal efficiencies.	Smart materials for TES systems.
[39]	Glauber’s Salt (Na_2_SO_4_·10H_2_O)/PAAS	MWCNTs	Mitigate leakage of the CPCM, increase thermal conductivity, analysis of other thermal properties.	Solar energy storage.
[40]	Magnesium nitrate hexahydrate (Mg(NO_3_)_2_·6H_2_O)	MWCNTs Graphite Nanoparticles	Enhancement of thermal charging/discharging, enhancement of thermal conductivity.	Solar energy storage.
[41]	Sodium Acetate Trihydrate (SAT)	Aluminum Nitride	Study thermal conductivity, phase change temperature, and latent heat for mass fractions of 3%, 4%, and 5%.	Research, none other specified.
[42]	Calcium Chloride Hexahydrate (CaCl_2_·6H_2_O)	Al_2_O_3_ TiO_2_ Cu SiO_2_	Thermal and physical property analysis.	Thermal energy storage.
[43]	Sodium Thiosulfate Pentahydrate	CNTs Graphite Nanoparticles (GNPs)	Thermal conductivity enhancement, charging/discharging time	Thermal energy storage.
[44]	Sodium Acetate Trihydrate/Carboxymethyl Cellulose	Silver Nanoparticles	Latent heat enhancement, phase change, temperature and supercooling analysis.	Thermal energy storage.

**Table 2:** Basic PCMs and their thermophysical properties before and after nano enhancement.

Ref.	PCM	Nanoparticle	Nanoparticle wt%	Thermal Conductivity [W/mK]	Latent Heat [kJ/kg]	Phase Change Temperature [°C]
[22]	Paraffin	nano-graphite	0%	0.1264	209.33	28.81
			1%	0.3650	202.58	27.73
			4%	0.4971	193.26	27.5
			7%	0.5685	183.62	27.66
			10%	0.9362	181.81	27.8
[23]	Palmitic acid	Carbon nanotubes (CNTs)	0%	0.22	208.0	62.4
			0.2%	6–7% ↑	200.4	62.1
			0.5%	12–13% ↑	197.7	62.3
			1%	46% ↑	184.0	61.1
[24]	n-octadecane	TiO_2_ (Titanium Dioxide)	0%	~0.47	-	28
			1%	~0.56	-	-
			3%	~0.85	-	-
			5%	~0.57	-	-
[25]	Water based Nanofluid	Multi wall CNTs	0%	0.61	-	Evaluated at T = 30
			0.15%	0.648	-	-
			0.3%	0.685	-	-
			0.45%	0.697	-	-
			0.6	0.71	-	-
[26]	n-octadecane	Alumina (Al_2_O_3_)	0%	0.13	243.1	26.5
			5%	0.133	225.6	26.0
			10%	0.14	212.3	26.3
[27]	Stearic acid	TiO_2_ (Titanium Dioxide)	0%	0.19	128.65	~53
			0.05%	0.23	128	~53.5
			0.1%	0.25	127.3	~53.8
			0.15%	0.283	126.2	~54
			0.2%	0.30	125.6	~55
			0.25%	0.31	124.8	~55.5
			0.3%	0.325	124.2	~55.8
[28]	Hexadecane	Graphite nanoplatelets	0%	0.668	232.41	18.65
			3%	0.992	217.33	18.27
			5%	1.161	-	-
	Octadecane		0%	0.497	241.97	28.91
			3%	0.873	240.92	29.96
			5%	0.999	-	-
	Paraffin Wax		0%	0.356	142.72	54.38
			3%	0.454	140.99	55.91
			5%	0.616	-	-
[38]	Hydrated Salts	Graphene oxide	0%	0.68	189.1	-
			NA	-	200.3	-
			0.5%	0.74	-	-
			1%	0.82	-	-
			1.5%	0.94	-	-
			2%	1.05	-	23
			2.5%	-	-	-
			3%	1.1	-	-

**Table 3:** Comparison of thermal properties of paraffin/TiO_2_ NEPCM with and without sodium stearoyl lactylate (SSL) before and after 80 thermal cycles [60].

SSL Included	Cycle	Mass Fraction wt%	Thermal Conductivity [W/m °C]	Latent Heat [J/g]	Phase Change Temperature [°C]
No	0	3%	0.18	136.4	60.64
Yes	0	3%	0.195	167	56
No	80	3%	0.131	134.71	58.34
Yes	80	3%	0.143	166.05	54.82

## Nomenclature

Al_2_O_3_	Aluminum Oxide
CB	Carbon Black
CNT	Carbon Nano Tubes
CPCM	Composite Phase Change Material
Cu	Copper
CuNWs	Copper Nanowires
CuO	Copper (II) Oxide
DSC	Differential scanning calorimeters
GNPs	Graphite Nanoparticles
GNSs	Graphite Nanosheets
MWCNT	Multi walled carbon nanotubes
NEPCM	Nano-enhanced phase change material
PAAS	Poly Acrylate Sodium
PAAAM	Poly(acrylamide-co-acrylic acid)
PCM	Phase Change Material
SEM	Scanning electron microscopy
SWCNT	Single walled carbon nanotubes
SAT	Sodium Acetate Trihydrate
TCA	Thermal conductivity analyzer
TEM	Transmission electron microscopy
Al_2_O_3_	Aluminum Oxide
Mg(NO_3_)_2_·6H_2_O	Magnesium nitrate hexahydrate
Na_2_SO_4_·10H_2_O	Glauber’s Salt
SiO_2_	Silicon Dioxide
TiO_2_	Titanium Dioxide
Q	Heat Transfer
a	Nanoparticle mass fraction
r	Nanoparticle radius
Cp	Specific heat
G	Gravitational acceleration
M	Mass
T	Temperature
T_m_	Melting Temperature
T_i_	Initial Temperature
T_f_	Final Temperature
V	Sedimentation velocity
ΔH	Change in enthalpy
η	Dynamic viscosity
ρ_pcm_	Density of phase change material
ρ_np_	Density of nanoparticles
